# Dermoscopic Rainbow Pattern in Deep Penetrating Nevus

**DOI:** 10.5826/dpc.1202a46

**Published:** 2022-04-01

**Authors:** Arturo Robles-Tenorio, Miriam Sarahí Preciado-Aguiar, Ricardo Quiñones-Venegas, Francisco Javier Salazar-Torres

**Affiliations:** 1 Escuela de Medicina y Ciencias de la Salud, Tecnológico de Monterrey, I. Morones Prieto 3000, Monterrey, México; 2 Instituto Dermatológico de Jalisco “Dr. José Barba Rubio”, Federalismo 3102, Zapopan, México

**Keywords:** nevus, dermoscopy, dermis, pigmentation, rainbow

## Introduction

The deep penetrating nevus (DPN) is a benign, acquired, melanocytic lesion that shows intense pigmentation and infiltration into the reticular dermis or subcutaneous tissue [1,2]. It affects young individuals before the third decade of life, primarily in the head and neck region. DPN usually presents as an asymptomatic, well-defined, symmetric, solitary, blue, brown, or black, papule or nodule. Due to its clinical and histopathological similarities, DPN is often confused with malignant melanoma, blue nevus, and Spitz nevus. Since dermoscopic images of DPN are scarce, its features are not well established. Here, we present a case of DPN in a patient with Fitzpatrick type V skin that showed the rainbow pattern under polarized immersion dermoscopy.

## Case Presentation

**Figure 1:**
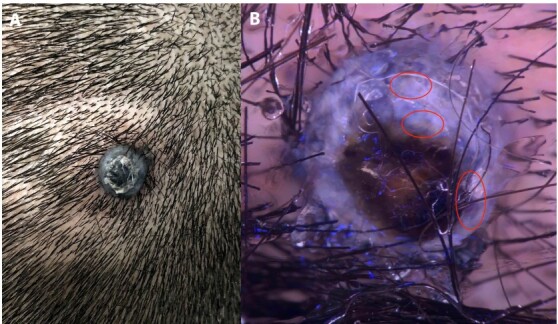
Deep penetrating nevus. (A) Clinical appearance. (B) Polarized immersion dermoscopy revealed a pigmented center, rainbow patterns (red ovals), and bluish-white structureless areas.

**Figure 2:**
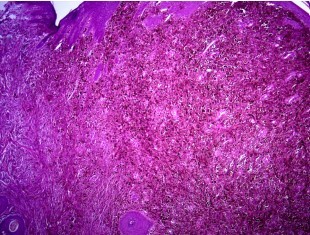
Histopathology of deep penetrating nevus. A symmetric tumor composed of spindle-shaped melanocytes extending to the deep dermis was observed (H&E × 40).

A 13-year-old male with Fitzpatrick type V skin presented with a 1-year history of an enlarging lesion on the scalp. On examination, there was an 8 × 5 × 5 mm, well-defined, black, hyperkeratotic nodule with a central erosion (Figure 1A). Polarized dermoscopy with ultrasound gel immersion showed a pigmented center surrounded by rainbow patterns and bluish-white structureless areas (Figure 1B). An excisional biopsy with a 3-mm margin was performed. On histopathology, a benign-appearing, symmetric tumor composed of epithelioid and spindle-shaped melanocytes extending to the hypodermis was observed (Figure 2), compatible with DPN. At the 24-month follow-up there was no recurrence.

## Conclusions

There are less than 5 dermoscopic descriptions of DPN, including a globular blue-brown pattern and a polychromatic appearance [1,2]. Polarized immersion dermoscopy is a suitable technique to evaluate nodular, melanocytic lesions, especially when hyperkeratosis, fissures, and ridges are present. The rainbow pattern and the clinical appearance of DPN in high Fitzpatrick skin types are rare findings among the available images from the literature. Increasing awareness of this condition in skin of color, as well as selecting an adequate dermoscopy technique can help to refine the characterization of DPN in underrepresented populations.
